# Efficacy of Metformin as Adjuvant Therapy in Metastatic Breast Cancer Treatment

**DOI:** 10.3390/jcm11195505

**Published:** 2022-09-20

**Authors:** Nourhan M. Essa, Heba F. Salem, Marwa O. Elgendy, A. Gabr, Mervat M. Omran, Nivin A. Hassan, Hanaa M. Tashkandi, Steve Harakeh, Marian S. Boshra

**Affiliations:** 1 Clinical Pharmacy Department, Faculty of Pharmacy, New Valley University, El-Kharja 72511, Egypt; 2 Department of Pharmaceutics and Industrial Pharmacy, Faculty of Pharmacy, Beni-Suef University, Beni-Suef 62521, Egypt; 3 Department of Clinical Pharmacy, Teaching Hospital of Faculty of Medicine, Faculty of Medicine, Beni-Suef University, Beni-Suef 62521, Egypt; 4 Department of Clinical Pharmacy, Faculty of Pharmacy, Nahda University (NUB), Beni-Suef 62764, Egypt; 5 Medical Oncology Department, South Egypt Cancer Institute, Assiut University, Assiut 71111, Egypt; 6 Pharmacology Unit, Cancer Biology Department, National Cancer Institute, Cairo University, Giza 11796, Egypt; 7 Pharmacology and Experimental Oncology Unit, Cancer Biology Department, South Egypt Cancer Institute, Assuit University, Assiut 71515, Egypt; 8 Department of General Surgery, School of Medicine, King Abdulaziz University, P.O. Box 80218, Jeddah 21589, Saudi Arabia; 9 King Fahd Medical Research Center, King Abdulaziz University, Jeddah 22254, Saudi Arabia; 10 Yousef Abdul Latif Jameel Scientific Chair of Prophetic Medicine Application, Faculty of Medicine, King Abdulaziz University, Jeddah 22254, Saudi Arabia; 11 Clinical Pharmacy Department, Faculty of Pharmacy, Beni-Suef University, Beni-Suef 62521, Egypt

**Keywords:** metformin, metastatic breast cancer (MBC), non-diabetic patients

## Abstract

Background: Metformin has been reported to have an anti-tumorigenic impact against metastatic breast cancer (MBC) cells through several mechanisms. Its effect can be evaluated by using many variables such as the response rate (RR) as well as the progression-free survival (PFS). Materials and methods: A prospective study was conducted to investigate and estimate the metformin effect on MBC. About 107 subjects were included in the study and were divided into two groups: Group A included non-diabetic MBC patients treated with metformin in conjunction with chemotherapy and group B included those treated with chemotherapy alone. Both PFS and RR were used as a criteria to evaluate the treatment outcome. Associated adverse effects of metformin were also assessed. Results: The average age of the participants in group A and group B was 50 vs. 47.5, respectively. No significant differences were detected between both cohorts concerning RR levels (regression disease (RD) 27.8% vs. 12.5%, stationary disease (SD) 44.4% vs. 41.7%, progression disease (PD) 27.8% vs. 45.8%, respectively, *p* = 0.074). Moreover, PFS showed no significant difference between both groups (*p* = 0.753). There was no significant correlation between metformin concentration and their adverse effects on the study participants. Conclusion: Metformin as an adjuvant therapy to MBC undergoing chemotherapy showed no significant survival benefit as determined by RR and PFS.

## 1. Introduction

Breast cancer (BC) is considered the most frequently diagnosed prevalent malignancy and cancer among women all over the world [1]. In Egypt, BC represents 19.3% of total primary malignant tumors, as reported by the Egyptian National Cancer Institute [2]. Th five-year survival rate of localized BC and MBC patients has been reported to be 99% and 27%, respectively [3]. Consequently, MBC remains an incurable disease, despite the survival of MBC patients is gradually improving. MBC is well-defined as a heterogeneous disease ranging from a solitary metastatic lesion to diffuse and spread to multiple organs [4]. The main goals of MBC treatment are to prolong the survival rate among patients and improve their quality of life [5].

It is evident that the insulin pathway is responsible for developing various human cancers, such as BC [6]. The anabolic effect of insulin, which leads to reaching DNA synthesis and proliferation of cells [7], can be attributed to insulin-like growth factors (IGF-1), which are the growth hormones’ endocrine mediators acting in an autocrine and paracrine manner in order to regulate apoptosis, differentiation, transformation, and cell growth in various tissues such as the breast [8]. It was found that IGF-1 involvement does not only promote proliferation but also cell growth via directly activating specific trans-membrane tyrosine kinase receptors that are primarily overexpressed in BC, resulting in the up-regulation of the insulin receptor-substrate-2 (IRS2) and causing a downstream activation in the in PI3K-Akt and MAPKinase pathways [9]. Furthermore, the IGF-1 has been shown to allow epithelial cancer cells to gain migratory access to mesenchymal cell characteristics after losing their polarity in a process referred to as the epithelial to mesenchymal transition (EMT), which is one of the significant processes in metastatic cascades [10].

**Figure 1:**
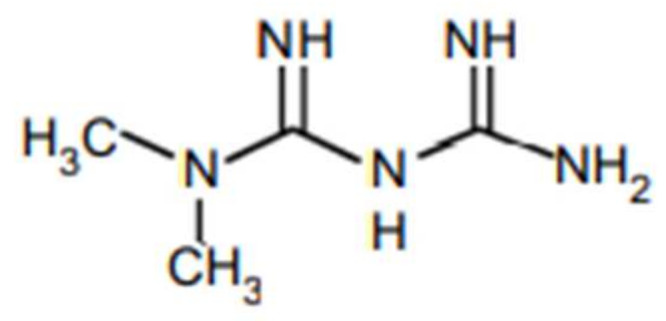
Molecular structure of metformin.

Several studies have revealed that metformin (N,N-dimethylbiguanide) (Figure 1) [11], which is a crucial anti-diabetic drug discovered by the French physician Jean Sterne [12], has an anti-tumorigenic impact on cancer cells through several mechanisms, including the indirect mechanism (insulin-dependent), in which metformin activates AMPK, which leads to the decrease of the gluconeogenesis process and the increase of glucose uptake in muscle cells, thereby resulting in the reduction of the blood glucose level and the insulin level. Decreasing the level of insulin in the blood inhibits the progression of cancer cells, as the higher level of insulin activates a higher number of insulin receptors on cancer cells, leading to the excessive growth of tumor cells [11,13,14,15,16,17]. In the direct mechanism (insulin-independent), metformin activates AMPK, which leads to the increased activation of TSC2 and reduced mTOR activity. The reduction in the mTOR activity causes the decrease in the level of some factors, including 4E-binding proteins and ribosomal protein s6 kinase; furthermore, it inhibits the protein synthesis as well as the progression of cells [11]. Other studies have indicated that the reduction of fatty synthase occurs through the activation of AMPK [11,17]. Furthermore, AMPK activates the acetyl coenzyme A carboxylase, which is mainly responsible for increasing the catabolism process of the cells [16].

Moreover, several studies have confirmed that metformin significantly contributes to inducing apoptosis and inhibiting the proliferation of cells through the interaction with the IGF pathway [18]. Consequently, it has been considered as a possible adjuvant to conventional therapy used in managing BC [19]. Moreover, metformin also alleviates BC cell resistance, thus triggering recent chemotherapeutic agents to function collaboratively with metformin [20].

A previous study on BC patients receiving neoadjuvant chemotherapy revealed that diabetic patients who were treated with metformin demonstrated a better response to treatment as compared to those diabetic patients receiving other types of anti-diabetic drugs, as well as non-diabetic BC cases [21]. Adding metformin to chemotherapy in patients with metastatic breast cancer improved the objective response rate compared to the placebo group but had no effect on the progression-free survival or overall survival in a recent meta-analysis study [22]. Based on the results of the current systematic and meta-analysis review, adding metformin to chemotherapy or endocrine therapy in non-diabetic MBC females does not improve RR, PFS, or OS compared to chemotherapy or endocrine therapy alone [22,23]. In addition, the hypothesis that metformin-based methods may be less beneficial in metastatic illness than in early stage cancer was highlighted in the phase I research of metformin plus erlotinib [24].

This study aimed to assess the effect of metformin as an adjuvant therapy with chemotherapy on RR and PFS in MBC patients.

## 2. Materials and Methods

### 2.1. Study Design

A prospective randomized study was conducted using metformin versus placebo in non-diabetic females with MBC under chemotherapy according to the fixed protocol at the South Egypt Cancer Institute (SECI), Assiut University. The study was carried out during the period extending from June 2020 to July 2021. One hundred and seven MBC female patients were included, divided into two groups: Group A (n = 57), treated with chemotherapy and metformin, and Group B (n = 50), treated with chemotherapy alone. This study aimed to assess progression-free survival (PFS) as well as the response rate (RR) in both groups.

All the studied patients were older than 18 years old, non-diabetic, and treated with chemotherapy. In contrast, males with BC, non-metastatic breast cancer patients, patients with more than one cancer (double cancer), patients on hormonal therapy or radiotherapy, diabetic patients, patients with a history of cardiac diseases, and patients with hypersensitivity or allergy to metformin were excluded from the study.

### 2.2. Treatment Schedule

Patients were divided into two groups: Group A included 57 patients who were subjected to a combination of metformin (500 mg twice daily) and chemotherapy according to the fixed protocol for six months, and Group B (control group) included 50 patients who received chemotherapy alone according to the fixed protocol for six months.

The treatment continued until the evidence of progressive disease, even if chemotherapy was altered or discontinued before the disease development, and when the patient experienced unacceptable toxicity.

The progression date was defined as the first day on which the conditions for the progressive disease occurred. In the case of non-measurable disease, the progression date was termed as the earliest new lesion appearance, symptoms deterioration, as well as increased disease burden significant enough to change the treatment.

### 2.3. Follow-Up Parameters

At baseline, the follow-up parameters were assessed as well as after six months (pre- and post-treatment), and computed tomography imaging was also carried out for the chest, abdomen, and pelvis to assess the tumor status. The tumor RR was evaluated using the RECIST 1.1 criteria for target lesions. Complete Response (CR) was represented as the disappearance of all target lesions and the measurement of short axis pathological lymph nodes must be reduced to less 10 mm. Partial Response (PR) was represented as a reduction in the sum of diameters of target lesions of at least 30% compared to the baseline. For Progressive Disease (PD), at least a 20% increase of at least 5 mm must be detected in the sum of diameters of the target lesions compared to the smallest sum in the study. Moreover, PD can be considered with the appearance of one or more new lesions. Finally, Stable Disease (SD) was reported when there was no sufficient shrinkage to consider for PR or sufficient increase to consider for PD compared to the smallest sum diameters in the study [25]. Some laboratory tests, such as kidney function, liver function, CBC, liver function tests, and tumor markers (CA15-3, CEA), were assessed during the period of treatment, and they were within normal. In addition, a toxicity profile was carried out during the period of follow-up. Moreover, many symptoms were evaluated, including heartburn, abdominal pain, physical weakness, flatulence, muscle pain, upper respiratory tract infection (URTI), hypoglycemia, diarrhea, weight loss, headache, unpleasant taste, nausea and vomiting, dyspnea, and anorexia.

#### 2.3.1. Blood Samples Collection

After six months of treatment, blood samples of about 2 mL were collected from all patients in Group A in EDTA tube after 2 h following the administration of metformin via vein puncture. The samples were allowed to coagulate and the plasma was separated by centrifugation at 4000 rpm for about 10–15 min, and the separated plasma was collected in Eppendorf tubes. Finally, they were kept at −80 °C, then plasma samples were transferred in ice containers for the assessment of the metformin dose level in the Pharmacology Unit, Tumor Biology Department at NCI, Cairo University.

#### 2.3.2. Assessment Method

An amount of 250 μL of patient plasma was placed in a glass tube for sample extraction. Then, 750 μL of acetonitrile was added. Tubes were vortexed for 1 min before centrifuging at 10,000 g for ten minutes at 4 °C. The supernatant was subsequently placed into HPLC auto-sampler vials, with a 10 μL injection into the LC-MS system.

**Figure 2:**
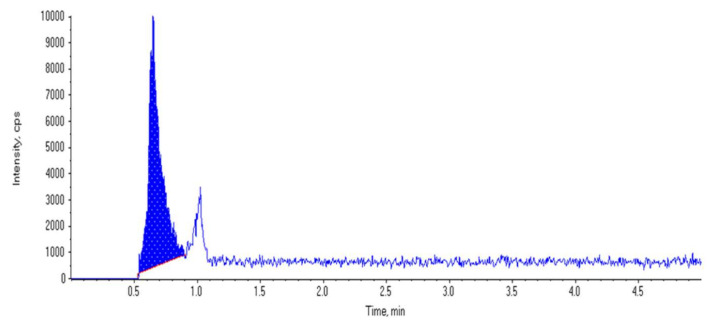
Chromatogram for detection of metformin standard with concentration 1.5 ug/mL.

The LC-MS-MS system was made using an electrospray ionization (ESI) interface coupled with an ABSCIEX Q TRAP 3200 mass spectrometer (ABSCIEX, Darmstadt, Germany), as well as an Agilent 1200 HPLC system (Agilent Technologies, CA, USA). The Analyst 4.0 software was utilized to collect data (ABSCIEX). The Agilent proshell EC, C18 (5 μm, 50 × 4.6 mm) reversed-phase analytical column was utilized for the separation (Agilent, CA, USA). The mobile phase, which was pumped at a 300 μL/ min flow rate, included formic acid (0.1%) in acetonitrile: water (40:60, *v*/*v*). The whole run interval was 5 min [26]. MRM was used to quantify the metformin utilizing curtain gas collision-induced dissociation, and after the transition of ions: m/z 130.2:60.1 for metformin. The chromatogram of the metformin is shown in Figure 2.

**Figure 3:**
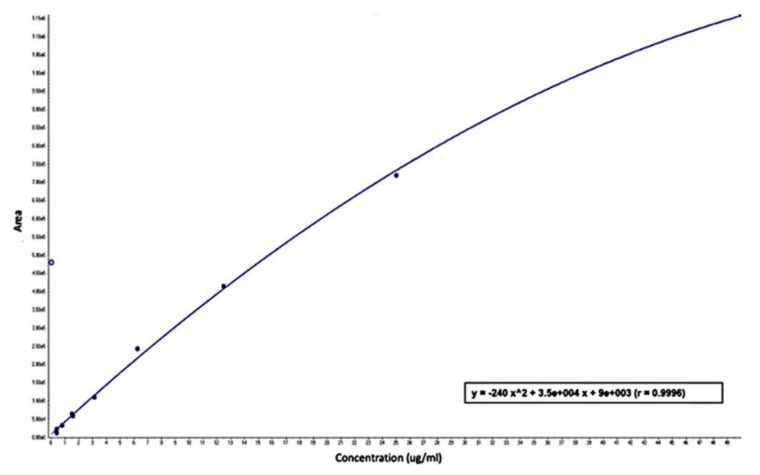
Calibration curve for metformin on concentration range (0.3–50 ug/mL). In the equation: x^2 = x^2^ and e= 10^3^.

To create a calibration curve, serial dilutions of standards were made in a drug-free medium at concentrations ranging from 0.3 to 50 ug/mL for the metformin, and extraction was performed as stated in the sample preparation, as shown in Figure 3.

### 2.4. Statistical Analysis

All statistical analyses were done using the 22nd version of the SPSS software. Data was expressed as mean ± standard deviation (±SD), frequencies (number of cases), and relative frequencies (percentages), when appropriate, or median and range when not normally distributed. Quantitative variable comparisons were made utilizing the Mann–Whitney U test for data with non-normal distribution, the Student *t*-test with data with normal distribution, and the Kruskal–Wallis test for comparing more than two continuous groups. Quantitative variable comparison was made utilizing the Student *t*-test for normally distributed data and the Mann–Whitney U test for data with non-normal distribution. Paired data with non-normal distribution (pre- and post-treatment) were compared using the Wilcoxon signed-rank test. Categorical data comparison was made utilizing the Chi-square (χ2) test. An exact test was utilized instead when the expected frequency was less than 5. The Kaplan–Meier test was used to compare the survival between the two groups. The *p*-values were one-tailed sets at a significance level of 0.05.

### 2.5. Ethics Statement

The Research and Ethics Committee (REC) of the Faculty of Pharmacy, Beni-Suef University (REC-H-PhBSU-21028), approved this study. Informed consent was obtained from all subjects involved in the study. Participation was voluntary. The confidentiality of the participants were completely guaranteed during the study. The study was carried out based on the good clinical practices and following the Declaration of Helsinki, as well as its amendments.

## 3. Results

A prospective study was conducted on 107 MBC patients who were collected from the Assiut University, Medical Oncology Department of South Egypt Cancer Institute (SECI), between June 2020 and July 2021.

The patients were categorized into two groups: Group A received chemotherapy and metformin (n = 57) and Group B received chemotherapy alone (n = 50). Both groups had received treatments until disease progression (6 months duration), unacceptable toxicity, and clinician decision based on response.

### 3.1. Demographic Data

Demographic data of both studied groups is presented in Table 1 and Table 2. The data demonstrated no statistically significant differences between the two groups. The average age of the patients in Group A was 49.56 ± 12.53 years and ranged from 26 to 75 years, while the average age in Group B was 48.40 ± 12.61 years and ranged from 27 to 83 years (*p* = 0.634). The majority of cases were diagnosed with invasive ductal carcinoma, which was the most common pathological type in both cohorts (98.2% vs. 94%, respectively, *p* = 0.338). According to the pathological grade, grade 2 was the most common among both groups at about 96.5% in Group A versus 92.0% in Group B, respectively (*p*-value = 0.415).

### 3.2. Response Rate (RR)

RR was assessed in Group A as well as Group B, and showed that (Table 3) patients with regression disease (RD) response was about 29.4% in Group A versus (12.8%) in Group B. Patients with stationary disease (SD) response were about 45.1% in Group A versus 42.6% in Group B. Patients with progression disease (PD) response were about 25.5% in Group A versus 44.7% in Group B. Metformin as adjuvant therapy did not help in improving the response as compared to the control group, with no substantial differences between Group A and Group B regarding the response (*p*-value = 0.068).

**Figure 4:**
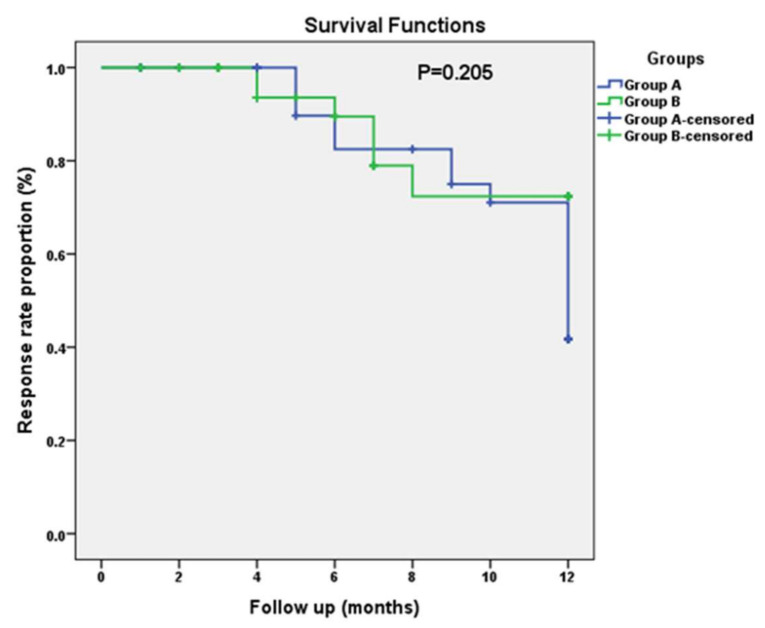
Kaplan–Meier curves for response rate among both studied groups.

The Kaplan–Meier curve for the response rate among both studied groups is represented in Figure 4. According to the Kaplan–Meier curve, during the median observation period of 12 months, the response rate was 41.8% in Group A versus 72.4% in Group B, with no significant difference between them (*p* = 0.205).

There was no significant change in the metformin concentration among the response status in the participants (*p-* value = 0.284), as shown in (Table 4).

### 3.3. Toxicity Profile of Metformin

The symptoms checklist was documented during the follow-up period. The side effects of metformin in Group A during the follow-up period are illustrated in Table 5. All patients received metformin as an adjuvant treatment with chemotherapy with tolerability without any threatened side effects interfering with the continuation of the treatment. It was shown that the most common symptoms were abdominal pain (36.8%), followed by diarrhea (33.3%), anorexia, nausea, and vomiting (24.6%), weight loss and heartburn (17.5%), dyspnea (14%), headache and unpleasant taste (12.3%), muscle pain (10.5%), physical weakness, flatulence and URTI (8.8%), and finally no patient suffered from hypoglycemia (0%). These symptoms lasted between 2 and 3 weeks, then resolved with time.

The adverse effects in patients receiving metformin as an adjuvant to CTH are presented in Table 6 in descending order. There was no significant correlation between metformin administration and the associated adverse effects among participants (Table 6).

### 3.4. Progression-Free Survival (PFS)

**Figure 5:**
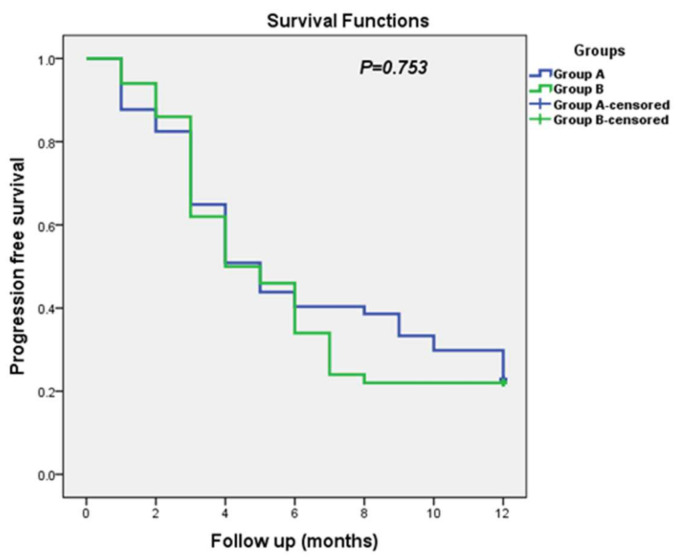
Kaplan–Meier curves for progression free survival among both studied groups.

PFS levels in the two groups during the one-year follow-up period are presented in Table 7. The median PFS for patients in Group A was 5 months versus 4 months in Group B, with no significant differences (*p* = 0.753). The Kaplan–Meier curve for the progression-free survival among both studied groups is represented in Figure 5.

## 4. Discussion

This prospective randomized controlled trial was conducted to evaluate the efficacy of metformin as an adjuvant therapy in the treatment of MBC patients in terms of RR and PFS. Both studied groups were matched to a great extent in terms of demographic characteristics data to reduce the effect of demographics on the outcome of the study.

The results of our study showed no beneficial effect of metformin when used in conjunction with chemotherapy based on the RR in MBC levels in the MBC patients. The results of this study were similar to those of a recent double-blind trial published by Pimentel et al., where they used metformin in conjunction with chemotherapy in a phase II randomized trial versus placebo in MBC on 40 patients who were randomized into two groups (22 metformin, 18 placeboes). They reported no significant effect of adding metformin as an adjuvant to chemotherapy on the levels of RR in the studied patients [27].

A previous study conducted on 122 non-diabetic females with HER2-negative MBC by Nanni et al. was done to assess the efficacy of metformin (M) when used as adjuvant to chemotherapy on MBC patients. Based on the RR levels, the authors observed no marked differences in the overall RR levels between the two studied groups (*p* = 0.901) [28]. Similar findings were reported by Fenn et al., where they evaluated the effects of the combination of metformin with erlotinib in patients in a phase 1 trial on cases with metastatic triple-negative breast cancer (mTNBC). The authors reported no objective responses in study group [24]. In another study, Goodwin et al. reported no significant differences in the RR levels in the metformin group versus the placebo group [29].

Additionally, in a meta-analysis study by Wu et al., it was stated that OS, as well as PFS, were not substantially enhanced in patients who were treated with metformin in parallel with standard conventional treatment as compared to the placebo group receiving standard treatment (OS: HR 1.02, 95% CI: 0.71–1.46, *p* = 0.916; PFS: HR 1.14, 95% CI: 0.86–1.50, *p* = 0.366) [30]. Recently, Rabea et al. reported that, in MBC patients, the mean PFS was 4.4 vs. 5.1 months in case of the control and metformin groups, respectively. Furthermore, the authors stated that metformin use did not significantly prolong OS or PFS in non-diabetic women patients with MBC [31]. Concerning the PFS levels that were documented in the present study during the 1-year follow-up period, no marked differences were observed in the median PFS between both studied groups. This revealed that metformin in combination with chemotherapy had no favorable impact on PFS. Similarly, Pimentel et al. reported that there was no significant effect of adding metformin as an adjuvant to chemotherapy either on PFS or overall survival (OS) [26]. When compared to control patients, Goodwin et al. found that metformin did not substantially extend OS or PFS in non-diabetic metastatic BC cases receiving metformin combined with conventional chemotherapy [29]. Moreover, Nanni et al. showed that the average PFS was 9.4 and 9.9 months in the metformin and placebo groups, respectively, and the same authors concluded that there is no evidence supporting the anticancer activity of M in combination with first-line CT in MBC [27].

Wang et al. (2019) investigated if metformin directly impacted the BC cells metastasis and proliferation. They reported that metformin plasma concentration in patients reached about 60 μM, and both 50 μM and 100 μM concentrations were evaluated for their efficacy in an in vitro setting. Their results indicated, based on in vitro data, that metformin had no effect on proliferation when one or two doses were used [32]. Several studies have shown that metformin has a strong therapeutic response in the prevention of the proliferation of tumors in the breast, lung, prostate, and colon [33,34,35,36]. Bowker et al. performed a study involving three groups of T2DM patients which used metformin, sulfonylurea, and insulin, and showed that the mortality rate was higher in the last two groups of patients compared to patients taking metformin. In their study, the occurrence of death per 1000 patients taking metformin, sulfonylurea, and insulin, who were under follow-up for one year, was 3.5, 4.9, and 8.8, respectively [13]. In another study performed on non-diabetic women with adjuvant (early-stage) breast cancer, metformin decreased the level of insulin by 22%. Moreover, Campagnoli et al. studied breast cancer in women with the use of metformin and found that it decreased the level of insulin and its damaging effects, such as insulin resistance [37]. A randomized double blind trial on MBC patients receiving standard chemotherapy with metformin vs a placebo showed an increase in the overall survival rate [11].

A recent meta-analysis by Wu et al. demonstrated that adding metformin to conventional therapy improved the objective response rate (ORR) by 30.3% (33/109) in the metformin plus standard treatment group and 16.1% (18/112) in the placebo group (RR 1.92, 95% CI: 1.19–3.10, *p* = 0.008) [30].

A randomized control clinical trial registered at clinicaltrials.gov (#NCT04143282) by Rabea et al. (2021) on 50 stage IV BC females reported that the radiologic response was substantially improved in the metformin group compared to controls [31].

In a meta-analysis study conducted by Wu et al., it indicted that the daily dose of metformin may impact the objective response rate (ORR). The authors reported that a 500 mg dose of metformin induced a better impact than using 1000 mg dose. As an anti-cancer agent, metformin mediated its activity via the AMPK/mTOR pathway. A prior trial revealed that metformin suppressed the signaling of mTOR in a dose-dependent manner, denoting that low-dose metformin significantly suppressed mTOR through the TSC and AMPK pathway, whereas the high-dose metformin might follow a different mechanism of action [30,38]. The study findings may in part demonstrate why a low metformin dosage resulted in a better impact than higher doses [30]. These differences among the different studies could be attributed to the dose of the metformin used, the site of use, and the number of metastasis originally present. In addition, the compliance rate of the studied participants could significantly contribute to evaluating the real effect of metformin in RR. Based on that, we should evaluate our studied cases and the outcome based on the consistency of patients receiving metformin to evaluate its real efficacy on RR, which is a limitation of our study.

To confirm the clinical anti-tumor effect of metformin, we compared the metformin concentrations according to the response of patients, and a trend was observed when higher metformin concentrations were used among patients who achieved RD than those patients with PD, but with no marked differences noted due to the small sample size of our participants. This highlights the need for conducting randomized studies using more patients to get a better indication related to the efficacy of metformin on the response status of MBC patients to therapy. Moreover, to try to define the best threshold of serum metformin levels to be used as an anti-tumor adjuvant therapy to achieve the best outcome.

All patients received metformin as an adjuvant treatment with chemotherapy with tolerability without any threatened side effects interfering with the continuation of the treatment. The tolerated abdominal pain and diarrhea were linked to the use of metformin as prevalent associated side effects, which was documented in 36.8% and 33.3% of our studied patients, respectively. One of the mechanisms that may induce a disruption in gut motility is related to either a decreased absorption rate of bile salts [39] or to a lower transport of serotonin within the gut, or both [40]. Metformin is characterized by a good safety profile because of its potential to reduce the risk of hypoglycemia and clinically relevant drug interaction, as noted by Rojas and Gomes, who reported fewer adverse effects or modifications in metformin efficacy when administered in combination with other drugs [41]. Additionally, Wu et al. 2020 stated that there were less common adverse events observed upon using metformin in conjunction with the standard MBC treatment, demonstrating the safety of using it in the treatment of MBC patients [30]. Consistent with our findings, Rabea et al. illustrated that grade 1 diarrhea was the most observed and tolerated adverse effect of metformin, experienced by 36% of our participants [31].

Pimentel et al. presented a toxicity profile of metformin based on data collected during the follow-up period of those on metformin as compared to the placebo group. The authors indicated that high grade (grade III or IV) events occurred in 10 (58.8%) placebo cases as well as 7 (31.8%) metformin. In case of lower grade events (I or II), 68.2% of metformin and 35.3% of placebo cases experienced one or more events, and gastrointestinal (GI) toxicity was the most common system organ class (SOC). Such a toxicity is mainly attributed to the administration of many chemotherapy regimens which are not related to metformin administration alone, as it is equally shown in both studied groups [26]. Moreover, Nanni et al. reported that no substantial differences were noted between the metformin group versus the placebo group regarding some adverse effects, such as the development of grade 3 and 4 neutropenia, which was observed in 54% of the metformin group versus 72% of the placebo group. Other toxicities related to metformin administration included grade 2 vomiting, experienced by 21% of the metformin group subjects versus 6% in the placebo group, grade 2 fatigue, experienced in 39% of the subjects using metformin group versus 11% in the placebo group, and grade 2 diarrhea, experienced by 9% of the metformin group subjects versus none in the placebo group [27].

## 5. Conclusions

In conclusion, this prospective study showed that using metformin as an adjuvant to chemotherapy in metastatic breast cancer patients had no beneficial effect on the RR and PFS, and further studies are required to confirm or exclude the results. A trend was observed when higher metformin concentrations were used among patients who achieved RD than those with PD, according to the response of the patients, but with no marked differences noted. Moreover, metformin use in this study was tolerable without any threatening side effects that may have caused the discontinuation of the treatment.

## 6. Limitations

Further studies should be carried out on a large number of patients using different doses of metformin, and their compliance to the drug should be followed up.

## Figures and Tables

**Table 1:** Baseline patient characteristics (n = 107).

Variable Name		Group A (CTH + M), n = 57		Group B (CTH Alone), n = 50		*p*-Value
**Age (years), Mean ± SD**		49.56 ± 12.53		48.40 ± 12.61		0.634 *
**Median (range)**		50 (26–75)		47.5 (27–83)		
**Pathology**	**(IDC)**	56	(98.2)	47	(94.0)	0.338 **
	**(ILC)**	1	(1.8)	3	(6.0)	
**Grade**	**Grade 2**	55	(96.5)	46	(92.0)	0.415 ***
	**Grade 3**	2	(3.5)	4	(8.0)	
**Menopausal status**	**Pre-menopausal**	24	(42.1)	29	(58.0)	0.101 **
	**Post-menopausal**	33	(57.9)	21	(42.0)	
**(BMI)**	**<25**	15	(26.3)	21	(42.0)	0.058 **
	**≥25–<30**	23	(40.4)	10	(20.0)	
	**≥30**	19	(33.3)	19	(38.0)	
**(ER)**	**Negative**	17	(29.8)	19	(38.0)	0.372 **
	**Positive**	40	(70.2)	31	(62.0)	
**(PR)**	**Negative**	25	(43.9)	20	(40.0)	0.687 **
	**Positive**	32	(56.1)	30	(60.0)	
**Her2neu**	**Negative**	30	(52.6)	30	(60.0)	0.444 **
	**Positive**	27	(47.4)	20	(40.0)	
**Luminal A**	**No**	33	(57.9)	26	(52.0)	0.541 **
	**Yes**	24	(42.1)	24	(48.0)	
**Luminal B**	**No**	40	(70.2)	41	(82.0)	0.155 **
	**Yes**	17	(29.8)	9	(18.0)	
**Her2neu overexpression**	**No**	47	(82.5)	39	(78.0)	0.563 **
	**Yes**	10	(17.5)	11	(22.0)	
**Triple negative**	**No**	51	(89.5)	45	(90.0)	0.929 ***
	**Yes**	6	(10.5)	5	(10.0)	
**CEA, Median (range)**		16.2 (0.5–142.0)		28.3 (1.9–06.0)		0.154 ****
**CA 15-3, Median (range)**		39.4 (7.1–241.8)		28.9 (4.3–413.7)		0.562 ****

Quantitative data are expressed as mean ± SD and median (range), qualitative data are expressed as a number (percent). M: metformin; CTH: chemotherapy; ILC: invasive lobular carcinoma; IDC: invasive ductal carcinoma; BMI: body mass index; PR: progesterone receptor; ER: estrogen receptor; CA 15-3: cancer antigen 15-3; CEA: carcinoembryonic antigen. *p*-value * by *t*-test, *p*-value ** by Chi-square (χ2) test, *p*-value *** by Exact test, *p*-value **** by Mann–Whitney U test.

**Table 2:** Site and number of metastasis, and the initial response of the studied participants (n = 107).

Variable Name		Group A (CTH + M), n = 57		Group B (CTH + P), n = 50		*p*-Value
**Site of Mets**	**Visceral**	34	(59.6)	25	(50.0)	0.317 #
	**Non-visceral**	23	(40.0)	25	(50.0)	
**No. of Mets**	**1**	14	(24.6)	23	(46.0)	0.067 #
	**2**	30	(52.6)	17	(34.0)	
	**>2**	13	(22.8)	10	(20.0)	

Visceral sites include adrenal glands, brain, liver, lungs, as well as pleura (with or without effusion). Non-visceral sites were defined as skin, bones, chest wall, lymph nodes, as well as the breast. Qualitative data are presented as a number (percent). Significance set at *p* < 0.05. *p*-value # by Chi-square (χ2) test.

**Table 3:** Response of the studied participants after 6 months of treatment (n = 98).

Variable Name		Group A (CTH + M), n = 51		Group B (CTH Alone), n = 47		*p*-Value
**Response**	**(RD)**	15	(29.4)	6	(12.8)	0.068 #
	**(SD)**	23	(45.1)	20	(42.6)	
	**(PD)**	13	(25.5)	21	(44.7)	

Qualitative data are expressed as a number (percent). Significance set at *p*< 0.05. *p*-value # by Chi-square (χ2) test. (RD): regression disease, (SD): stationary disease, (PD): progression disease.

**Table 4:** Metformin concentration according to the response status of the studied participants after 6 months of treatment (n = 51).

Variable Name		Mean ± SD	*p*-Value #
**Response**	**RD**	2.41 ± 0.52	0.284
	**SD**	2.13 ± 0.59	
	**PD**	2.11 ± 0.63	

Quantitative data are expressed Mean ±SD. Significance set at *p* < 0.05. *p*-value # by Kruskal–Wallis test.

**Table 5:** Adverse effects in patients receiving metformin as an adjuvant to CTH (n = 57).

Adverse Events	N	(%)
**Abdominal pain**	21	(36.8)
**Diarrhea**	19	(33.3)
**Nausea and vomiting**	14	(24.6)
**Anorexia**	14	(24.6)
**Weight loss**	10	(17.5)
**Heartburn**	10	(17.5)
**Dyspnea**	8	(14.0)
**Headache**	7	(12.3)
**Unpleasant taste**	7	(12.3)
**Muscle pain**	6	(10.5)
**Physical weakness**	5	(8.8)
**Flatulence**	5	(8.8)
**URTI**	5	(8.8)
**Hypoglycemia**	0	(0.0)

Qualitative data are expressed a number (percent), URTI: upper respiratory tract infection.

**Table 6:** The concentration of metformin according to the occurrence of its side effects (n = 51).

Side Effects		Metformin Concentration	
		Mean ± SD	*p*-Value #
**Heartburn**	No	2.19 ± 0.58	0.692
	Yes	2.09 ± 0.53	
**Abdominal pain**	No	2.19 ± 0.58	0.703
	Yes	2.14 ± 0.57	
**Physical weakness**	No	2.19 ± 0.58	0.634
	Yes	1.97 ± 0.33	
**Flatulence**	No	2.19 ± 0.57	0.502
	Yes	2.00 ± 0.61	
**Muscle pain**	No	2.16 ± 0.56	0.655
	Yes	2.26 ± 0.71	
**URTI**	No	2.16 ± 0.59	0.384
	Yes	2.31 ± 0.42	
**Hypoglycemia**	No	2.17 ± 0.57	-
	Yes	-	
**Diarrhea**	No	2.23 ± 0.58	0.290
	Yes	2.06 ± 0.55	
**Weight loss**	No	2.17 ± 0.56	0.881
	Yes	2.19 ± 0.64	
**Headache**	No	2.16 ± 0.55	1
	Yes	2.22 ± 0.73	
**Unpleasant taste**	No	2.19 ± 0.58	0.576
	Yes	2.05 ± 0.50	
**Nausea and vomiting**	No	2.17 ± 0.62	0.813
	Yes	2.18 ± 0.42	
**Dyspnea**	No	2.16 ± 0.59	0.637
	Yes	2.21 ± 0.43	
**Anorexia**	No	2.20 ± 0.62	0.835
	Yes	2.08 ± 0.38	

Quantitative data are presented as Mean ± SD. Significance defined by *p* < 0.05. *p*-value # by Mann–Whitney U test.

**Table 7:** PFS among both studied groups (n = 107).

Item	Median				Log Rank (Mantel–Cox) *p*-Value
	Estimate	Std. Error	95% Confidence Interval		
**Group A (CTH + Metformin)**	5.000	0.624	3.776	6.224	
**Group B (CTH + Placebo)**	4.000	0.758	2.515	5.485	0.753
**Overall**	5.000	0.514	3.992	6.008	

## Data Availability

Data is available on request.
